# Response of a Mu-class glutathione *S*-transferase from black tiger shrimp *Penaeus monodon* to aflatoxin B1 exposure

**DOI:** 10.1186/s40064-016-2381-4

**Published:** 2016-06-22

**Authors:** Yun Wang, Lihui Liu, Jianhua Huang, Yafei Duan, Jun Wang, Mingjun Fu, Heizhao Lin

**Affiliations:** Key Laboratory of Aquatic Product Processing, Ministry of Agriculture, South China Sea Fisheries Research Institute, Chinese Academy of Fishery Sciences, Guangzhou, 510300 People’s Republic of China; Pearl River Fisheries Research Institute, Chinese Academy of Fishery Sciences, Guangzhou, 510380 Guangdong Province People’s Republic of China

**Keywords:** *Penaeus monodon*, Mu-class glutathione *S*-transferase, Aflatoxin B1 exposure, cDNA cloning

## Abstract

Glutathione *S*-transferases (GSTs) are a family of multifunctional phase II enzymes that are involved in the detoxification of exogenous and endogenous compounds. In this study, a full-length cDNA of Mu-class GST (*PmMuGST*) was isolated from the hepatopancreas of *Penaeus monodon* using rapid amplification of cDNA ends method. The full length cDNA of *PmMuGST* is 867 bp, contains an open read frame of 660 bp, and encodes a polypeptide of 219 amino acids with a molecular mass of 25.61 kDa and *p*I of 6.15. Sequence analysis indicated that the predicted protein sequence of *PmMuGST* was very similar to (86 %) that of *Litopenaeus vannamei*. A conserved domain of GST_N_Mu_like (PSSM: cd03075) and GST_C_family_superfamily_like (PSSM: cl02776) was indentified in *PmMuGST*. Real time quantitative RT-PCR analysis indicated that *PmMuGST* was present in all of the tested tissues. *PmMuGST* transcripts both in the hepatopancreas and in the muscle were significantly induced after 14 days of treatment with a low dosage of AFB1 (50 μg/kg) exposure and were significantly inhibited after 42 and 56 days of a high dosage of AFB1 (1000, 2500 μg/kg AFB1) exposure. Taken together, the Mu-class GST from *P. monodon* was inducible and was involved in the response to AFB1 exposure.

## Background

To counteract the harmful effects of endogenous and exogenous compounds, such as drugs and mycotoxins, living organisms employ an array of enzymatic detoxification processes that have been classified into three distinct phases (Bathige et al. 2014). Enzymes in phase I and II convert xenobiotics to less toxic water-soluble metabolites, which are later eliminated from the cell via phase III enzymes (Sheehan et al. 2001). Glutathione S-transferases (GST_S_ E.C. 2.5.1.18) are the major enzymes in phase II metabolism and are involved in the detoxification process by catalyzing the conjugation of glutathione (GSH) with substrates that have an electrophilic carbon, nitrogen or sulfur atom. The substrates include halogenonitrobenzenes, arene oxides, quinones, α- and β-unsaturated carbonyls and aflatoxins (Hayes et al. 2005). Based on the substrate specificity, antibody cross-reactivity and sensitivity to inhibitors, GSTs from various organisms have been identified as representing at least 15 different classes (Wang et al. 2013). To date, many classes of GSTs have also been cloned and characterized in several aquatic species including the Pacific oyster *Crassostrea gigas* (Boutet et al. 2004), rock shell *Thais clavigera* (Rhee et al. 2008), disk abalone *Haliotis discus discus* (Wan et al. 2008a), yellow catfish *Pelteobagrus fulvidraco* (Ku et al. 2014) and Manila clam *Ruditapes philippinarum* (Bathige et al. 2014). Regarding aquatic crustaceans, Contreras-Vergara et al. (2004) reported the first Mu-class of GSTs in the white shrimp *Litopenaeus vannamei*. GSTs, as components of the detoxification mechanism in *L. vannamei*, function similarly to what has been observed in mammals and other invertebrates (Salazar-Medina et al. 2010; Goncalves-Soares et al. 2012). Two classes of GSTs (*FcMuGST, FcThetaGST*) were cloned from Chinese shrimp *Fenneropenaeus chinensis*, and the *FcMuGST* transcript was determined to be increased in response to *Vibrio anguillarum* infection, while *FcThetaGST* showed little change at the transcript level. It was speculated that *FcMuGST* may play a vital role in the detoxification process after *V. anguillarum* infection (Ren et al. 2009b). The theta GST from freshwater prawn *Macrobrachium rosenbergii* was thought to play a variety of functions such as in immune responses and detoxification (Arockiaraj et al. 2014). Generally, GSTs play vital roles in the detoxification and immune system of aquatic animals, and many studies have undertaken biochemical characterization and transcription profiles of GSTs after pathogenic and toxic chemical exposures. However, there is minimal information regarding the molecular structure of GSTs and their response to AFB1 from the black tiger shrimp.

The black tiger shrimp *P. monodon* is a major globally farmed species that requires a diet high in protein. Due to the high cost of fishmeal and environmental conservation concerns, people are seeking vegetable origin feedstuffs to replace fishmeal used in aqua feeds; however, feed with high concentration of plant ingredients, such as peanut, corn, soybean and rice bran are more susceptible to mycotoxin contamination (Zychowski et al. 2013). Mycotoxins are toxic metabolites produced naturally by certain fungal species that represent an unavoidable problem due to their presence in globally consumed cereals (Marroquín-Cardona et al. 2014; da Rocha et al. 2014). Among all of known mycotoxins, aflatoxins are the most investigated and are classified as group Ι carcinogen by International Agency for Research on Cancer (IARC, 1994). Four major aflatoxins (AFB1, AFB2, AFG1 and AFG2) have been reported as direct contaminants in feed ingredients and formulated diets (Raghavan et al. 2011). It was reported that AFB1 caused abnormalities such as poor growth, low apparent digestibility, physiological disorder and histological changes principally in the hepatopancreas of *L. vannamei* (Ostrowski-Meissner et al. 1995; Tapia-Salazar et al. 2012) and *P. monodon* (Boonyaratpalin et al. 2001; Gopinath and Raj 2009; Gopinath et al. 2012). However, the underlying metabolism and detoxification mechanisms of AFB1 in *P. monodon* warrants further investigation.

Previous studies have demonstrated that GSTs play a crucial role in the detoxification of AFB1 by catalyzing the conjugate formation of *exo*-AFB1-8,9-epoxide–GSH (AFBO-GSH) (Rawal et al. 2010) and are considered a major factor in the determination of species sensitivity to AFB1 (Hayes et al. 1991; Dohnal et al. 2014; Tulayakul et al. 2005). To elucidate the function of GSTs and their roles in the defense mechanisms of *P. monodon* against AFB1, the aims of this study were to clone and characterize a Mu-class GST cDNA from *P. monodon* and to investigate the mRNA and protein accumulation after *P. monodon* exposure to AFB1 within 8 weeks.

## Methods

### Diet preparation and dietary treatments

Six experimental diets containing 0 (D0), 50 (D1), 100 (D2), 500 (D3), 1000 (D4), and 2500 (D5) μg/kg AFB1 (Sigma Chemicals, St. Louis, MO, USA) were used to assess the toxic effects of AFB1 on black tiger shrimp *P. monodon*. The formulation and approximate composition of the six experimental diets were shown in Table 1. The diets were prepared according to the method described by Niu et al. (2008). All ingredients were ground in a laboratory grinder and sifted using a 60 μm sieve. Raw material of each of the components of the experimental diets were weighed, combined and thoroughly mixed to homogeneity in a Hobart-type mixer. Next, oil was added and thoroughly mixed for 5 min. Deionized water (30 % of the dry ingredient mixture) was added and mixed until the consistency was suitable for pelleting. The wet mixture was extruded in a monoscrew extruder (Institute of Chemical Engineering, South China University of Technology, Guangzhou, China) through a 1.0 mm die. The resulting pellets were steamed in an electric oven at 90 °C for 40 min for starch gelatinization and then dried at 25 °C with the aid of an air conditioner and an electrical fan. All of the resulting diet was stored at −20 °C until feeding.

**Table 1:** Ingredients and nutrient composition of the basal diet (g/100 g dry matter)

Ingredient	%
White fish meal^a^	31
Soybean meal^b^	16.3
Peanut bran^b^	17
Wheat flour^b^	19
Beer yeast^b^	5
Squid meal^b^	3
Soybean lecithin^c^	2
Fish oil^d^	1
Soybean oil^a^	1
Choline chloride (50 %)	0.6
Monocalcium phosphate	1
Ascorbic phosphate ester^e^	0.1
Vitamin premix^f^	1
Mineral premix^g^	1
Sodium alginate	1

Proximate composition of each diet^h^ (g/kg)						
Group^i^	D0	D1	D2	D3	D4	D5
Aflatoxin B1^j^ (μg/kg)	0	50	100	500	1000	2500
Crude protein	47.31	47.26	47.35	47.61	47.44	47.56
Lipid	8.93	7.79	8.41	8.27	8.01	8.65
Ash	11.46	11.43	11.66	11.64	11.7	11.83
Moisture	9.65	9.80	9.75	9.71	9.75	9.61

^a^Imported from N.E.L.T.O. Australia Pty Ltd

^b^Zhuhai Shihai Feed Corporation Ltd, Zhuhai, China

^c^Kemin Industries (Zhuhai) Ltd., Zhuhai, China

^d^Imported from New Zealand (Bakels Edible Oils Ltd, Mt Macnganui)

^e^Guangzhou Chengyi Company Ltd., Guangzhou, China

^f^Vitamin premix (g/kg): h-Carotene, 3 M.I.U.; Cholecalciferol, 0.6 M.I.U.; Thiamin, 3.6; Riboflavin, 7.2; Pyridoxine, 6.6; Cyanocobalamine, 0.02; a–Tocopherol, 16.5; Menadione, 2.4; Niacin, 14.4; Pantothenic acid, 4; Biotin, 0.02; Folic acid, 1.2; Inositol, 30; Ascorbic acid, 100; cellulose was used as a carrier

^g^Mineral premix (g/kg): P, 120; Ca, 120; Mg, 15; Fe, 1.5; Zn, 4.2; Cu, 2.1; K, 75; Co, 0.11; Mn, 1.6; Se, 0.01; Mo, 0.005; Al, 0.025; I, 0.4; cellulose was used as a carrier

^h^Measured values

^i^Group: D0, 0 μg/kg AFB1; D1, 50 μg/kg AFB1; D2, 100 μg/kg AFB1; D3, 500 μg/kg AFB1; D4, 1000 μg/kg AFB1; D5, 2500 μg/kg AFB1

^j^Aflatoxin B_1_ was purchased from Sigma (St. Louis, MO, USA)

### Experimental animals and culture

Shrimp were obtained from a semi-intensive culture pond at Shenzhen Base, South China Sea Fisheries Research Institute of Chinese Academy of Fishery Sciences (Shenzhen, Guangdong), and all shrimp were fed with the basal diet (D0) for one week to acclimate to the experimental diets and conditions. A total of 540 healthy shrimp with an initial body weight of 1.15 ± 0.02 g were randomly allocated to 18 fiberglass tanks (800 L, 0.5 m^2^ bottom areas, 30 shrimp per tank), with three tanks total being fed with each one of the six diets. Each tank contained 600 L of sand-filtered seawater and was covered with a plastic mesh lid to prevent the shrimp from jumping out. The water was continuously aerated with two air stones. During the feeding trial, the range of water salinity and temperature was 37–38 g/L and 28–30 °C, respectively. All shrimp in each tank were initially fed daily with 6 % of their total body weight and were hand-fed to apparent satiation three times daily (8:00, 17:00 and 22:00). During the feeding trial, the amount of diet administered was progressively altered and adjusted according to the appetite of the shrimp by checking the excess feed at the bottom of the tanks after feeding for 1 h. Thus, overfeeding was minimized, and shrimp were fed close to satiation. The feeding trial lasted for 56 days.

### Sample collection and preservation

Hepatopancreas and muscle tissue of three shrimp from each tank were randomly sampled at 14, 28, 42 and 56 days and were immediately divided into two parts to quantify *PmMuGST* mRNA and analyze PmMuGST protein accumulation. All samples were stored in liquid nitrogen. At the end of the feeding trial, shrimp were fasted for 24 h and then weighed to measure growth performance. The growth performance and survival of *P. monodon* of all groups were calculated using the following equations:

$$\begin{aligned} {\text{Weight}}\;{\text{ gain }}\left( {{\text{WG}},\% } \right) & = \left( {{\text{mean}}\;{\text{final}}\;{\text{body}}\;{\text{weight}}{-}{\text{mean}}\;{\text{initial}}\;{\text{body}}\;{\text{weight}}} \right)/{\text{mean}} \\ & \quad \;\;{\text{initial}}\;{\text{body}}\;{\text{weight}} \times 100 \\ \end{aligned}$$

$${\text{Survival }}\left( \% \right) = {\text{final}}\;{\text{number}}\;{\text{of}}\;{\text{shrimp}}/{\text{initial}}\;{\text{number}}\;{\text{of}}\;{\text{shrimp}} \times 100$$

### RNA isolation and cDNA synthesis

Total RNA was extracted from the hepatopancreas and muscle tissues using TRIzol reagent (Invitrogen, Carlsbad, CA, USA) according to the manufacturer’s protocol. DNA contamination was removed from the RNA using RQ1 RNase-Free DNase (Promega, Medisen, WI, USA). Hepatopancreas and muscle RNA was used as the template for first-strand cDNA synthesis using M-MLV reverse transcriptase (Promega, Medisen, WI, USA) following the manufacturer’s instruction.

### PCR and cloning of *PmMuGST* cDNA

Full-length *PmMuGST* cDNA was obtained using reverse-transcription polymerase chain reaction (RT-PCR), and the 3′, 5′ rapid amplification of cDNA ends (RACE) method. Two pairs of degenerate primers (Table 2) were designed to clone a partial sequence of *PmMuGST*, based on the highly conserved nucleotide sequence of Mu-class GST from *L. vannamei* (GenBank accession no. AY573381), *F. chinensis* (Ren et al. 2009b) and *Rattus norvegicus* (GenBank accession no. NM_017014). PCR amplification was performed using the cDNA template from the hepatopancreas. The first RT-PCRs were conducted as follows: 5 min at 94 °C for one cycle followed by another 30 cycles of 30 s at 94 °C, 30 s at 60 °C, 30 s at 72 °C, and a final extension for 10 min at 72 °C followed by cooling to 4 °C. The second nested RT-PCR program was consisted of one cycle of 94 °C for 5 min, and another 30 cycles of 94 °C for 30 s, 55 °C for 1 min, 72 °C for 1 min followed by a 10 min extension at 72 °C. A partial *PmMuGST* cDNA fragment of 172 bp was obtained from two pairs of degenerate primers.

**Table 2:** Oligonucleotide primers used in this study

Primers	Sequences (5′–3′)	Sequence information
MuGST-F1	CCTACGAGATCTTCGACCAGCACCT	Degenerate primers
MuGST-R1	CYYCTYCAYTTCRTATYTCTTCCTCT	Degenerate primers
MuGST-F2	CAGGCTTTCCAGAAGAGGTTTG	Nested degenerate primers
MuGST-R2	GATCGTAAACTGAGCGTACTTGTTGC	Nested degenerate primers
PmMuGST-F1	CAGGAAGTACATGGCGTCCCCGGATTTC	3′ RACE PCR
PmMuGST-F2	GTAGATGGCTTGGTTTATGAAGAGGAAGA	3′ RACE-nested PCR
PmMuGST-R1	TTCTTCCTCTTCATAAACCAAGCCATCTAC	5′ RACE PCR
PmMuGST-R2	CCTGATGAAATCCGGGGACGCCATGTAC	5′ RACE-nested PCR
EF1A-F	AGTATGCTCCTTTTGGACGTTTTGC	Real-time PCR
EF1A-R	CCTTTTCTGCGGCCTTGGTAGTC	Real-time PCR
PmMuGST-F	ACGGGCACTGAGTACGAGGAGAAG	Real-time PCR
PmMuGST-R	GGCAGATTTGGGAAAGCGAGG	Real-time PCR
MuGST-EF	**CG** **GGATCC**ATGGTGCCTGTCCTGGG	Recombinant expression
MuGST-ER	**CCC** **AAGCTT**TCATTTTCCCTCAGCGATC	Recombinant expression

F and R stand for forward primers and reverse ones, respectively. MuGST-EF and MuGST-ER containing flanking non-complementary sequences (bold type) and the restriction sites (underlined). Y = C or T, R = A or G

Based on the partial sequence data of *PmMuGST*, the 3′ and 5′ ends were obtained using a SMART™ RACE cDNA Amplification Kit (Clontech, Otsu, Shiga, Japan). The primers used for cloning the full-length cDNAs of *PmMuGST* are listed in Table 2. For the first 3′ RACE, the PCR was performed as follows: one cycle of 94 °C for 3 min; 5 cycles of 94 °C for 30 s, 72 °C for 3 min; 5 cycles of 94 °C for 30 s, 70 °C for 30 s, and 72 °C for 3 min; 25 cycles of 94 °C for 30 s, 68 °C for 30 s, 72 °C for 3 min; and finally cooled to 4 °C. For the second 3′ end RACE, the reaction was carried out with UPM and a 3′ nested PCR primer (Table 2) using the first PCR product under the following conditions: one cycle of 94 °C for 3 min; 25 cycles of 94 °C for 30 s, 68 °C for 30 s, 72 °C for 3 min. The gene-specific primer PmMuGST-R1 (Table 2) and UPM were applied to perform the first 5′ RACE, which PCR conditions were the same as that used for the first 3′ RACE. The second 5′ RACE condition using the gene-specific primer PmMuGST-R2 and nested UPM, which PCR conditions were the same as that of the second 3′ RACE.

The amplified PCR products were resolved in a 2.0 % agarose gel and the target PCR fragment was purified using the Wizard SV Gel and PCR clean-up System (Promega, Madisen, WI, USA). The purified fragments were then ligated into the pMD18-T vector (TaKaRa, Otsu, Shiga, Japan) and used to transform *Escherichia coli* cells. The recombinant bacteria were identified by blue/white screening method and confirmed by PCR. Plasmids containing the insert were purified by Pure Yield™ Plasmid Midiprep System (Promega, Madisen, WI, USA) and used as the template for DNA sequencing.

### Analysis of nucleotide and amino acid sequences

The nucleotides and deduced amino acid sequences of *PmMuGST* cDNA were analyzed and compared using the BLAST search programs (NCBI, http://www.ncbi.nlm.nih.gov/BLAST/). The signal peptide was predicted by SignalP program (http://www.cbs.dtu.dk/services/SignalP/). The element of secondary structure of the deduced peptide from the clones was predicted by GOR4 software on the website (http://www.expasy.org/). The multiple sequence alignment of *PmMuGST* amino acid sequence was performed using the programs of Vector NTI advance 10.3 (Invitrogen). The CD-Search service was used to identify the conserved domains (CDs) present in predicted protein sequences against NCBI’s Conserved Domain Database (CDD, http://www.ncbi.nlm.nih.gov/Structure/cdd/cdd.shtml.) Phylogenetic tree and molecular evolutionary analysis were conducted using MEGA version 6 (Tamura et al. 2013).

### Tissue distribution of *PmMuGST* mRNA

To investigate the basal (control) mRNA level of *PmMuGST* in various tissues, total RNA was extracted from eight tissues, including hemocytes, hepatopancreas, muscle, heart, ovary, stomach, eyestalk and intestine, from three healthy *P. monodon* using Trizol regent (Invitrogen, UAS). The amount of *PmMuGST* mRNA in the different tissues was determined by quantitative real-time PCR (RT-PCR). The RNA samples were analyzed in 1.0 % agarose electrophoresis and quantitated at 260 nm, and all OD_260_/OD_280_ ratios were between 1.8 and 2.0. Total RNA (1 μg) was reverse transcribed using the PrimeScript™ Real time PCR Kit (TaKaRa, Otsu, Shiga, Japan) for real-time quantitative RT-PCR analysis. Elongation factor 1-alpha (EF1A) of *P. monodon* (GenBank accession no. GU136229) was used as an internal control.

### Recombinant expression of PmMuGST in *E. coli*

The primers for MuGST-EF and MuGST-ER (Table 2) contain flanking non-complementary sequences (bold type) so that the desired restriction sites (underlined) are included in the amplicons. The primers were added to the PCR reaction mix to amplify the MuGST cDNA fragments, which encoded the mature MuGST protein. PCR reactions of 10 μL consisted of 2 μL cDNA template, 0.4 μL each of 10 μM forward and reverse primer, 1 μL 10 × PCR Buffer, 1 μL 2.5 mM dNTPs, 0.5 μL Taq DNA polymerase (5 U/μL) LA and 4.7 μL PCR-grade H_2_O. The mixture was denatured at 95 °C for 3 min followed by 35 cycles of 95 °C for 30 s, 60 °C for 30 s and 72 °C for 1 min. The resulting PCR products (660 bp) containing the complete MuGST cDNA were digested with *BamH*I–*Hind*III restriction enzymes and then cloned into the pET28a(+) vector (Novagen, Darmstadt, Germany). Prior to cloning, the vector was also digested with *BamH*I–*Hind*III enzymes. Thus, a coding region for the N-terminal His_6_ tag was fused to the MuGST open reading frame. The correct sequence and in-frame insertion location of the insert were verified by DNA sequencing. The recombinant plasmids used to transform *E. coli* BL21 (DE3) (MerckMillipore, USA) and then induced with IPTG following the previously reported method (Wang et al. 2011). Finally, the synthesis of the recombinant protein was demonstrated by SDS-PAGE (12 % separating gel and 5 % stacking gel).

### Recombinant protein purification, polyclonal antibody preparation and western blot analysis

The culture volume was increased to 300 mL to obtain more recombinant protein. The transformed *E. coli* was incubated at 37 °C for 4 h at 200 rpm after inducing with 1 mM IPTG. The *E. coli* cells were harvested by centrifugation at 4 °C, and washed with ice-cold phosphate-buffer saline (PBS, pH 7.4) for three times, and then were sonicated for 30 min discontinuously (stopped from 10 s to 10 s to allow cooling of the sample) at 100 W on ice. After centrifugation (×10,000*g*) at 4 °C for 30 min, the supernatant was used for the purification of the recombinant protein through metal affinity chromatography (TALON Resin, Clontech TALON^®^ Superflow™ Metal Affinity Resin).

A polyclonal antiserum against PmMuGST was subsequently obtained from rabbits by injecting 1 mg of the purified recombinant protein with complete Freund’s adjuvant once and incomplete Freund’s adjuvant (Sigma) at a 1-week interval (Nadala and Loh 1998; Sahul Hameed et al. 1998). The rabbits were injected four times. The antiserum was collected from the rabbits at the end of the week after the last boost. Cell free protein extract from transformed bacteria was resolved in SDS-PAGE (12 % separating gel and 5 % stacking gel) and transferred to Immobilon membranes (MerckMillipore, USA). A protein that reacted with an anti-PmMuGST antibody (1:1000) and a horseradish peroxidase-conjugated goat anti-rabbit IgGs secondary antibody (1:2500) (Pierce, Thermo Fisher Scientific, Rockford, USA) was detected in the western blot analysis.

### Quantification of *PmMuGST* mRNA by real-time PCR

The mRNA level of *PmMuGST* in the hepatopancreas and muscle of *P. monodon* in response to AFB1 exposure were analyzed using a quantitative real-time PCR method, as previously described (Livak and Schmittgen 2001; Bergallo et al. 2010) (ABI StepOne Plus, Applied Biosystems, Foster City, CA, USA). A pair of gene-specific primers (PmMuGST-F and PmMuGST-R) was designed to amplify a product with 133 bp. A pair of primers of EF1A-F and EF1A-R (Table 2) was used to amplify a 120 bp length of fragment of EF1A mRNA as an internal control to verify the successful reverse transcription and to calibrate the cDNA template. Nuclease-Free water was used in the place of cDNA templates as a negative control. All samples were repeated in triplicate (n = 3). The qRT-PCR amplifications were carried out in a total reaction volume of 20 μL containing 10 μL 2 × Master Mix (Fermentas K0223, Thermo Fisher Scientific, Ottawa, ON, Canada), 0.8 μL cDNA, 0.3 μL each of 10 μM forward and reverse primer and 8.6 μL pcr-grade water. The real-time PCR program consisted of 95 °C for 10 min, followed by 40 cycles of 95 °C for 15 s, 60 °C for 30 s, and 72 °C for 15 s. Melting curve analysis of the amplified products was performed at the end of each reaction to confirm that only one PCR product was amplified and detected. The fluorescent real-time PCR data were analyzed using 7500 System SDS Software (Applied Biosystems, Foster City, CA, USA).

### Analysis of PmMuGST protein in the hepatopancreas by western blotting after AFB1 exposure

Hepatopancreas tissues of three *P. monodon* from each tank were collected at 14, 28, 42, and 56 days and were ground in liquid nitrogen, and RIPA lysis buffer (Boyotime, China) was added. The homogenates were centrifuged (12,000*g*, 30 min) at 4 °C to collect the supernatants. Total protein concentrations in each shrimp tissue extract were determined using a Thermo BCA™ protein assay kit. Equal amount of protein (30 μg) was added per lane of the gel for SDS-PAGE and western blotting analysis. The protein extracts were separated by 12 % SDS-PAGE and transferred onto PVDF membranes (MerckMillipore, USA). The membranes were blocked for 2 h at room temperature in the Blocking solution (5 % w/v skim milk, 1 × TBS, 0.1 % Tween-20), and then treated with anti-PmMuGST antibodies diluted in the Blocking solution (1:300) at 4 °C with gentle shaking overnight. After washing in TBS (24.23 g/L Tris–HCl, 80.06 g/L NaCl, pH 7.6) for three times, the membranes were incubated for 1 h at room temperature with horseradish peroxidase-conjugated goat anti-rabbit IgGs secondary antibody (1:1000) (Pierce, Thermo Fisher Scientific, Rockford, USA). Enhanced chemiluminescence substrates (Pierce, Thermo Fisher Scientific, Rockford, USA) were subsequently added to the membranes for 5 min. Each membrane was exposed to X-ray film (Uvipro) after removing the substrates according to the manufacturer’s instruction. Films were exposed to the membranes from 1 to 30 s initially and then adjusted exposure time depending on the signal intensity. After the exposure, X-ray films were removed from X-ray holder and quickly immersed into the developer. When the bands appeared, the development was immediate terminated. And the films were immersed in the fixer until were transparent. The films dried at room temperature. The optical density of the bands was determined directly on the film using IPP6.0 Analysis software. The relative intensity of objective protein was calculated based on the optical density ratio of PmMuGST and GAPDH band. GAPDH protein was used as an internal control. The amounts of GAPDH were also assessed to monitor the equal loadings of protein extracts.

### Statistical analysis

Data were expressed as the mean ± standard deviation (SD) unless otherwise indicated. The significant differences between groups were analyzed using one-way ANOVA. If significant differences were indicated at the 0.05 level, then Duncan’s Multiple Range comparison tests were applied to identify significant differences among treatments. The linear relationships among PmMuGST (relative *PmMuGST* mRNA expression levels and relative PmMuGST protein levels), AFB1 concentrations and times were tested using the General Linear Models procedure (GLM) of SPSS 16.0. Differences were considered to be significant at *P* < 0.05.

## Results

### Growth performance and survival of *P. monodon*

Shrimp fed with diets containing AFB1 (50–100 μg/kg) had lower WG as than D0 group (control), but there were no significant differences (*P* > 0.05). Moreover, shrimp fed with diets containing AFB1 (500–2500 μg/kg) exhibited significantly lower WG compared with the control group (*P* < 0.05). There were no significant differences in shrimp survival among all groups after feeding for 56 days (Table 3).

**Table 3:** Growth performance of *P. monodon* after dietary AFB1 stress for 56 days (*n* = 3)

Group	D0	D1	D2	D3	D4	D5
WG	320.18 ± 28.22^c^	293.76 ± 10.27^bc^	287.23 ± 25.04^bc^	272.37 ± 13.64^b^	265.45 ± 25.35^b^	90.46 ± 13.14^a^
Survival	73.33 ± 6.67	72.22 ± 3.85	75.56 ± 1.93	72.22 ± 9.62	65.56 ± 1.93	66.67 ± 6.67

Values are expressed as the mean ± SE of three replicates. The different lowercase letters represented the significant difference in the different group (*P* < 0.05)

Group: D0, 0 μg/kg AFB1; D1, 50 μg/kg AFB1; D2, 100 μg/kg AFB1; D3, 500 μg/kg AFB1; D4, 1000 μg/kg AFB1; D5, 2500 μg/kg AFB1

Weight gain (WG, %) = (mean final body weight − mean initial body weight)/mean initial body weight × 100

Survival (%) = final number of shrimp/initial number of shrimp × 100

### Analysis of the *PmMuGST* sequence and the predicted protein

**Fig. 1:**
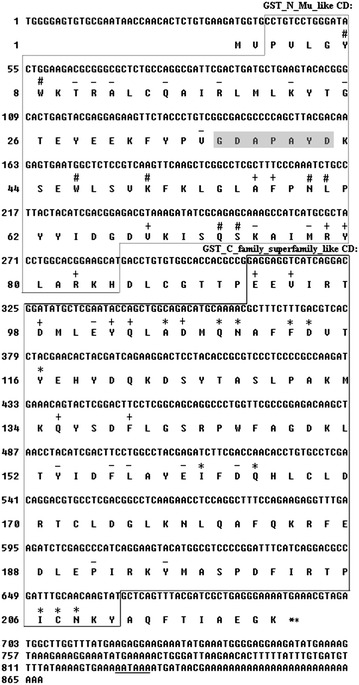
Nucleotide and deduced amino acid sequences of *PmMuGST* cDNA. The nucleotides are numbered from the first base at the 5*′*-end. The amino acids are numbered from the initial methionine. An open reading frame of 660 nucleotides encoding 219 amino acids contains the polyadenylation signal (*underlined*) and the stop codon (marked with *double asterisks* **). The organization of the predicted conserved domains (CD) using the CD-Search service are framed, including the GST_N_Mu_like CD and the GST_C_superfamily_like CD. The GSH-binding sites (G-site) in the N_terminal marked with “#”. The sites of substrate binding pocket (H-site) in the C_terminal were marked with “*”. The interacting interface sites of the N_terminal domain with the C_terminal domain are marked with “−”. The dimmer interface sites (GenBank accession number KM023785) are marked with “+”. The Mu loop (GDAPAYD) in the amino acid sequence is *shaded in gray*

The full-length 867 bp *PmMuGST* cDNA of *P. monodon* (GenBank accession number KM023785) includes an open reading frame of 660 bp encoding 219 amino acid residues, a 3*′* non-coding region of 173 bp with a polyadenylation signal (AATAAA) and a poly(A) tail (Fig. 1). Signal P analysis did not predict any signal peptide sequence in *PmMuGST*. The calculated molecular mass and p*I* were 25.61 kDa and 6.15, respectively. The full-length cDNA sequence of *PmMuGST* was first reported in this study and showed similarity to the GST sequence of *L*. *vannamei*. The predicted amino acid sequence of *PmMuGST* was found to be similar to the proteins of GST class Mu when analyzed by BLASTp with a creditable expectation value (E value ≤ 10^−3^). In a search of the CDs using the CD-Search service against NCBI’s CDD, the predicted protein sequence of *PmMuGST* cDNA was matched to CDs of the GST_N_Mu_like (PSSM: cd03075) and GST_C_family_superfamily_like (PSSM: cl02776) (Fig. 1). Additionally, the predicted protein sequence of *PmMuGST* cDNA also contains a G-site (from Pro^3^ to His^84^) that binds the GSH in the N-terminal region and an H-site (from Glu^92^ to Tyr^210^) that is a substrate binding site in the C-terminal (Fig. 1). In the GST_N_Mu_like domain, the secondary structural elements were arranged in a βαβαββα conformation. Similar to other Mu-class GSTs, the *PmMuGST* amino acid sequence also possessed a Mu loop between β2 and α2. The position of the seven amino acid Mu loop was located at residues 36–42 (GDAPAYD), which was shorter compared to those of mammals (Fig. 1). GST_C_family_superfamily_like domain consisted of four long and one short α helices and a short β sheet. The major stabilization factor in the G-site of Mu class GSTs is Tyr7 (Y) (Blanchette et al. 2007), which was also found in the sequence of *PmMuGST* (Fig. 1). As in the other GSTs, *PmMuGST* contains the conserved G-site motif FPNLPYYIDGD between the residue 57 and 67 (Contreras-Vergara et al. 2004).

### Homology analysis

A BLSATP search revealed that the *PmMuGST* amino acid sequence had an overall similarity of 42–86 % with Mu class GSTs of other species (Table 4). *PmMuGST* exhibited the highest similarity (86 %) with an *L. vannamei* GST and the lowest similarity with a Mu class GST of *Fasciola hepatica* (Table 4).

**Table 4:** Comparison of predicted *PmMuGST* amino acid with mu GST of other species

GenBank number	Species	Similarities (%)
AAT76663	*Litopenaeus vannamei*	86
AGJ70295	*Macrobrachium nipponense* GST	57
AFM86755	*Callorhinchus milii* Mu3 GST	56
ACO14549	*Esox lucius* Mu3 GST	56
NP_001103586	*Danio rerio* GST	55
NP_058710	*Rattus norvegicus* Mu1 GST	55
ABD67509	*Cyprinus carpio* Mu GST	55
P15626	*Mus musculus* Mu 2 GST	53
P46439	*Homo sapiens* Mu5 GST	51
P20136	*Gallus gallus* mu GST	49
P31670	*Fasciola hepatica* mu GST	42

**Fig. 2:**
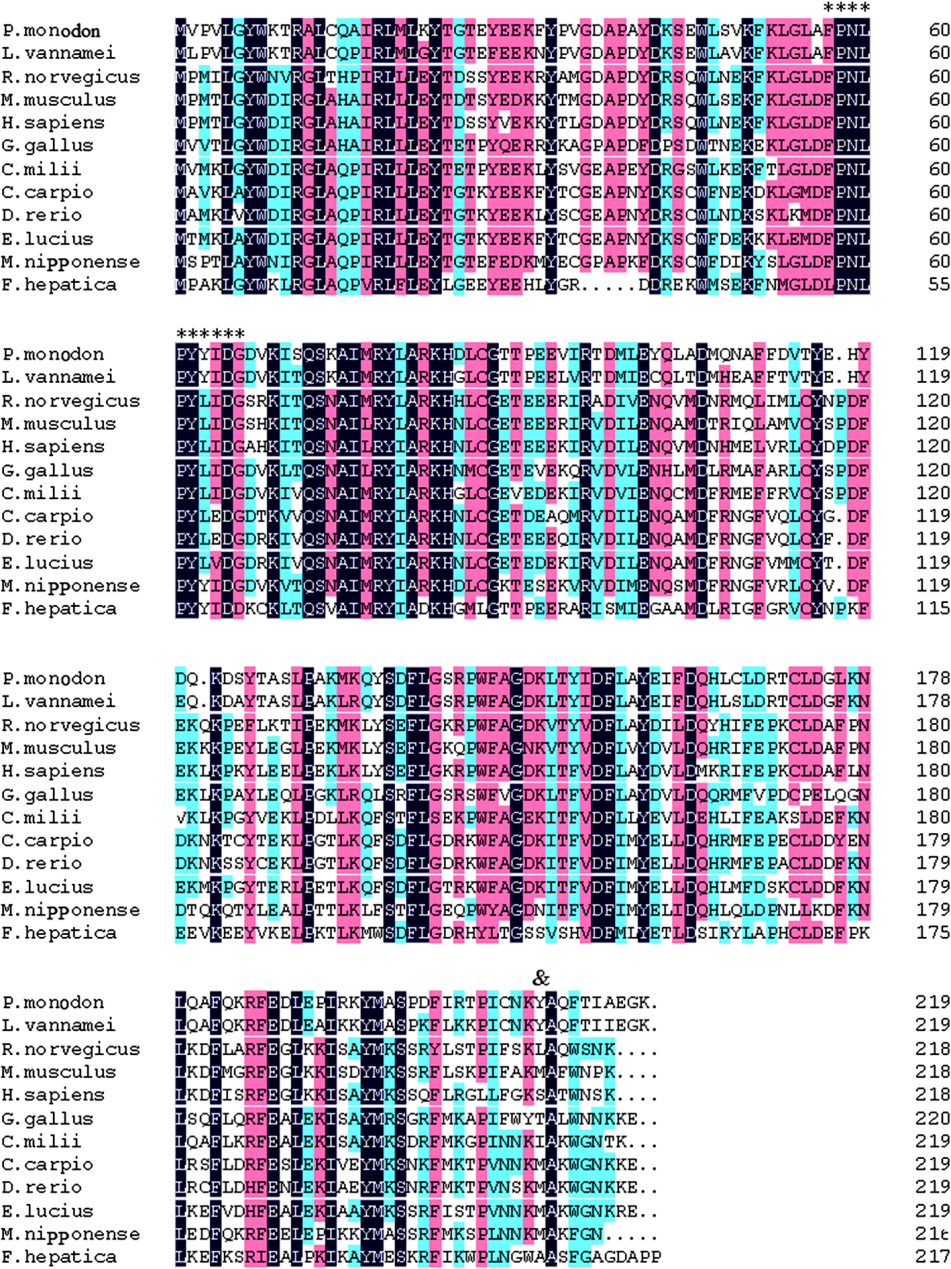
Multiple alignments of the deduced amino acid sequences of the *PmMuGST* with corresponding counterparts deposited in GenBank: *Litopenaeus vannamei* (AAT76663), *Rattus norvegicus* (NP_058710), *Mus musculus* (P15626), *Homo sapiens* (P46439), *Gallus gallus* (P20136), *Callorhinchus milii* (AFM86755), *Cyprinus carpio* (ABD67509), *Danio rerio* (NP_001103586), *Esox lucius* (ACO14549), *Macrobrachium nipponense* (AGJ70295), *Fasciola hepatica* (P31670). The G-site region is labeled with *asterisks*. The position of the residue equivalent to position 210 in other GSTs is labeled with “&”. The *black shaded region* indicates positions where all sequences share the same amino acid residue

Multiple alignment analysis of *PmMuGST* with other known GSTs revealed that the N-terminal region of all of the GSTs was highly similar and conserved, while the C-terminal was relatively diverse (Fig. 2). The numbers of consensus amino acid sequences that are shaded in black in the N-terminal region (from 3 to 84 bp) are higher than those in the C-terminal region (from 92 to 210 bp). Each GST is known to contain a G-site that binds the GSH substrate in its N-terminal and an H-site that binds xenobiotic compounds in the C-terminal (Armstrong 1997). All of the members of the highly diverse GST super family are capable of binding the tripeptide GSH; thus, it has been suggested that the structural features of the G-site might share a highly conserved amino acid sequence (Ren et al. 2009a). The H-site that binds xenobiotic compounds is the primary structure accounting for specificity and activity of GSTs, and it lacks amino acid sequence similarity to the G-site (Sheehan et al. 2001).

**Fig. 3:**
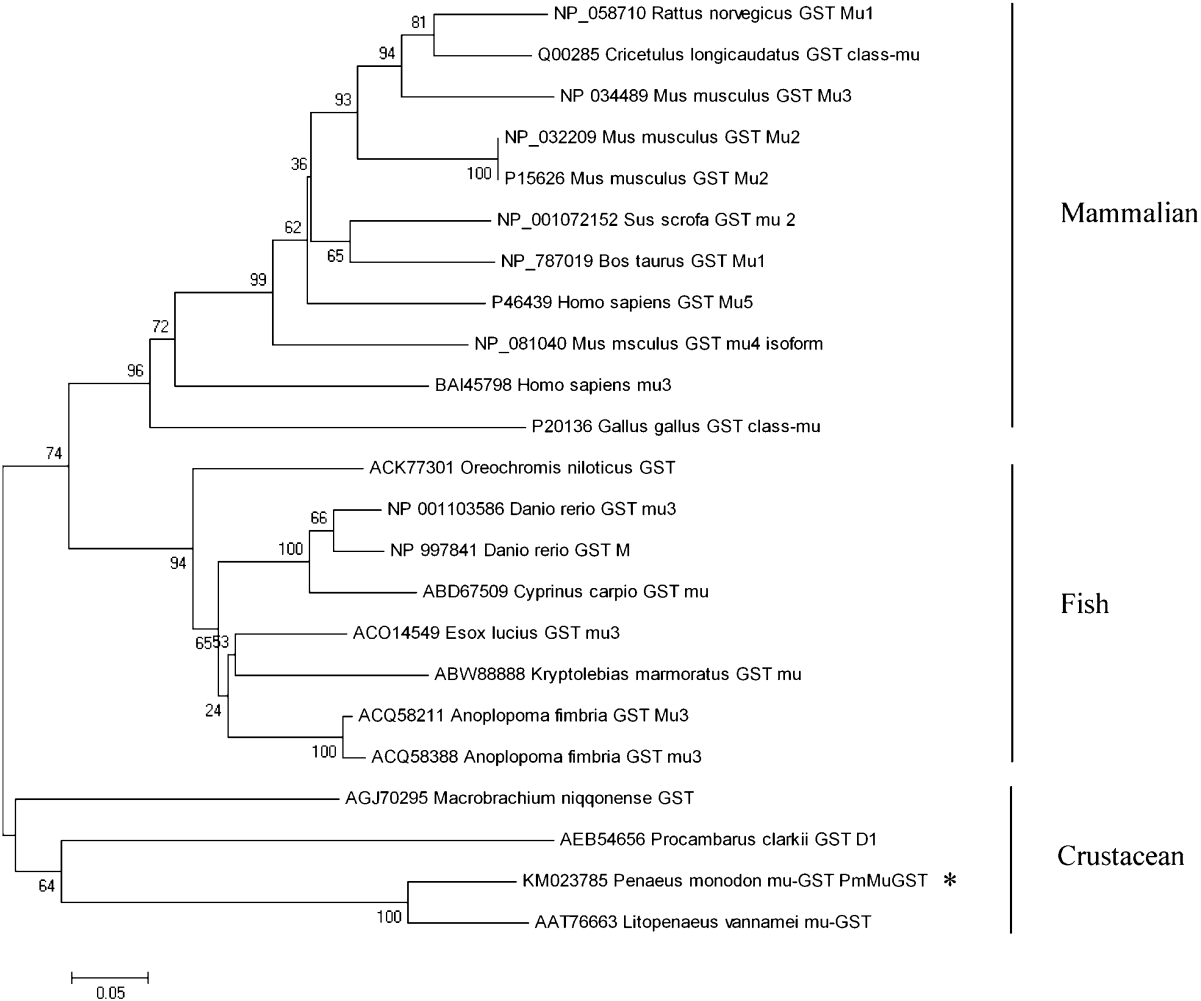
Phylogenetic tree constructed using the neighbor-joining method (MEGA v4.0) based on the deduced amino acid sequences from *PmMuGST* and other known GST Mu-class cDNAs from GenBank. The *PmMuGST* is labeled with an *asterisk* (*). *Numbers* next to the *branches* indicate the bootstrap value of *each internal branch* in the phylogenetic tree nodes from 1000 replicates

The phylogenetic tree was constructed using the neighbor-joining distance method in MEGA version 6.0. The known GSTs were classified into two groups (Fig. 3): one was GST class-Mu of crustaceans and the second one was GST class-Mu of mammalian and fish. The deduced amino acid of *PmMuGST* was in the same group as the GST from *L. vannamei*, *Procambarus clarkii* and *M. nipponense*. This group differed from the other groups containing the mammalian and the fish GST class-Mu subgroup, such as the mammalian GST class-Mu subgroup of *R. norvegicus*, *Cricetulus longicaudatus*, *M. musculus*, *Sus scrofa*, *Bos taurus*, *H. sapiens* and *G. gallus*, and the fish class-Mu subgroup of *Oreochromis niloticus*, *D. rerio*, *C. carpio*, *E. lucius*, *Kryptolebias marmoratus* and *Anoplopoma fimbria*. *PmMuGST* belongs to the crustacean GST class-Mu and was more closely related to the GST class-Mu of *L. vannamei* and *P. clarkii* than to that of *M. nipponense*. These results strongly support a common evolutionary lineage for shrimp GST class-Mu.

### Tissue distribution of *PmMuGST* mRNA

**Fig. 4:**
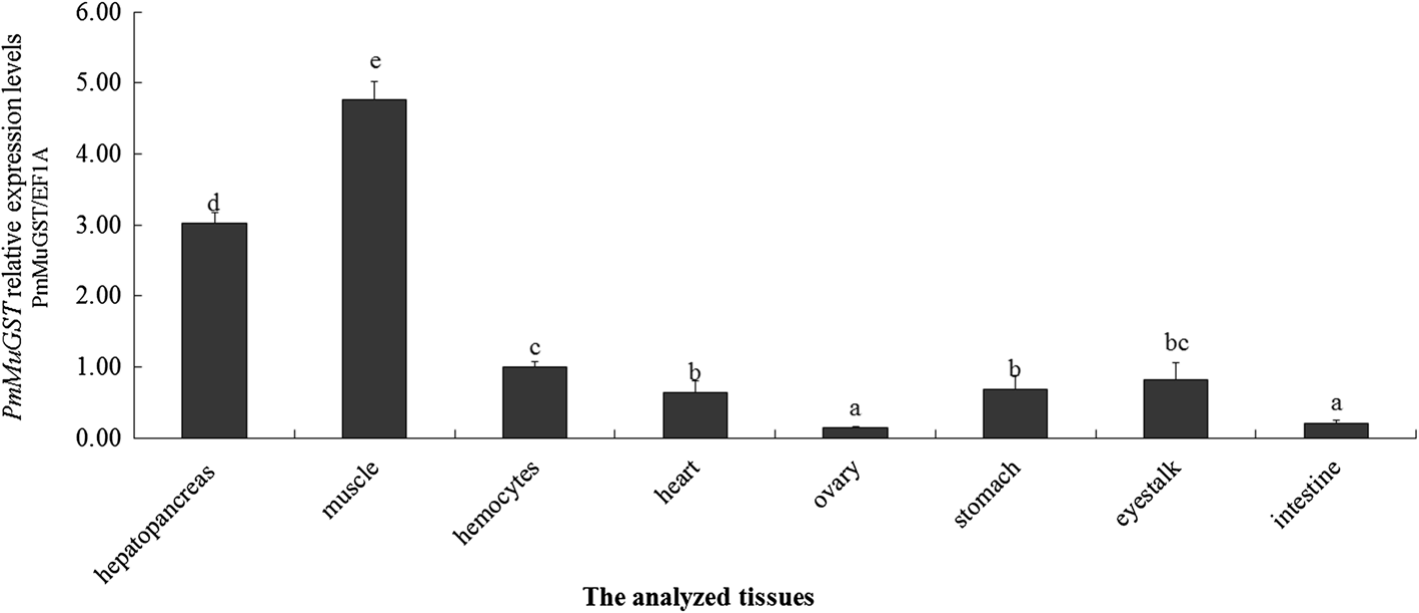
Tissue distribution of *PmMuGST* mRNA in *P. monodon*. qRT-PCR was performed with RNA from the hepatopancreas, muscle, hemocytes, heart, ovary, stomach, eyestalk and intestine samples of three healthy shrimp. Each sample was run in triplicate. The elongation factor 1-alpha (EF1A) gene was used as an internal control to calibrate the cDNA template for all the samples using the method described in the previous papers. *Vertical bars* represent the mean ± SD (*n* = 3). One-way ANOVA was used to compare different tissue distribution of expression levels. The *different lowercase superscripts* represent significant differences in pairwise comparisons with control group (*P* < 0.05)

The tissue-specific distribution of the basal (control) *PmMuGST* mRNA was determined by quantitative real-time RT-PCR. *PmMuGST* mRNA was found in all sampled tissues. As shown in Fig. 4, the maximum quantity of *PmMuGST* mRNA was found in the muscle, followed by the hepatopancreas, hemocytes, eyestalk, stomach, heart, intestine, and ovary.

### Purification of the PmMuGST recombinant protein and western blot analysis

**Fig. 5:**
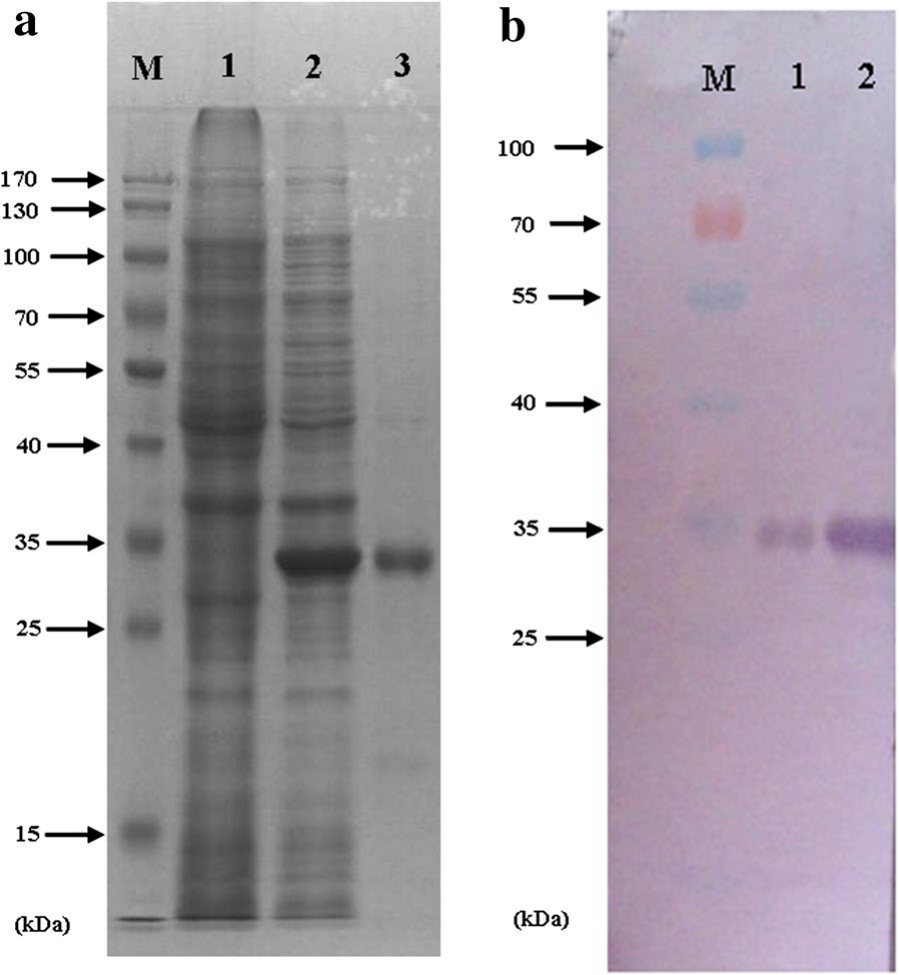
Expression and purification of the recombination *PmMuGST* fusion protein. Equal amounts of proteins (30 μg) were subject to SDS-PAGE and western blotting analysis. **a** Protein samples were separated by SDS-PAGE and stained with Coomassie Brilliant Blue. *Lane M*, protein standard; *lane 1*, crude extract of BL 21 (DE3) without plasmid; *lane 2*, crude extract of the transformed BL21 (DE3) with recombined pET28a (+) plasmid induced with IPTG; *lane 3*, purified *PmMuGST* fusion protein. **b** Protein samples were analyzed by immunoblotting with anti-*PmMuGST* antibody. *Lane M*, protein standard; *lane 1*, crude extract of the transformed BL 21 (DE3) with recombined pET28a (+) plasmid induced with IPTG; *lane 2*, purified *PmMuGST* fusion protein

The recombinant expression vector pET28a was transformed into the BL21 (DE3) strain and was over expressed as a His_6_-tagged protein after IPTG induction for 4 h. SDS-PAGE analysis revealed that the PmMuGST recombinant protein was very prominent on the stained gel (Fig. 5a). The PmMuGST recombinant protein was purified using the metal affinity chromatography. The recombinant protein contained the amino acid of the expression plasmid pET28a (+), which included a His-tag (HHHHHH) and a T7-tag (MASMTGGQQ). The calculated molecular mass of recombinant protein was 29.02 kDa and was larger than that of the mature PmMuGST (Fig. 5a). A band of approximately 35 kDa in size corresponding to the His-tag PmMuGST recombinant protein was observed after IPTG induction and was found to react with the anti-PmMuGST antibody. No bands were found in the same position in the BL21 (DE3) without the plasmid (Fig. 5a). The recombinant protein PmMuGST was successfully recognized with a rabbit anti-PmMuGST antibody (Fig. 5b).

### Expression profiles of *PmMuGST* in the hepatopancreas and muscle of *P. monodon* after exposure to AFB1

**Fig. 6:**
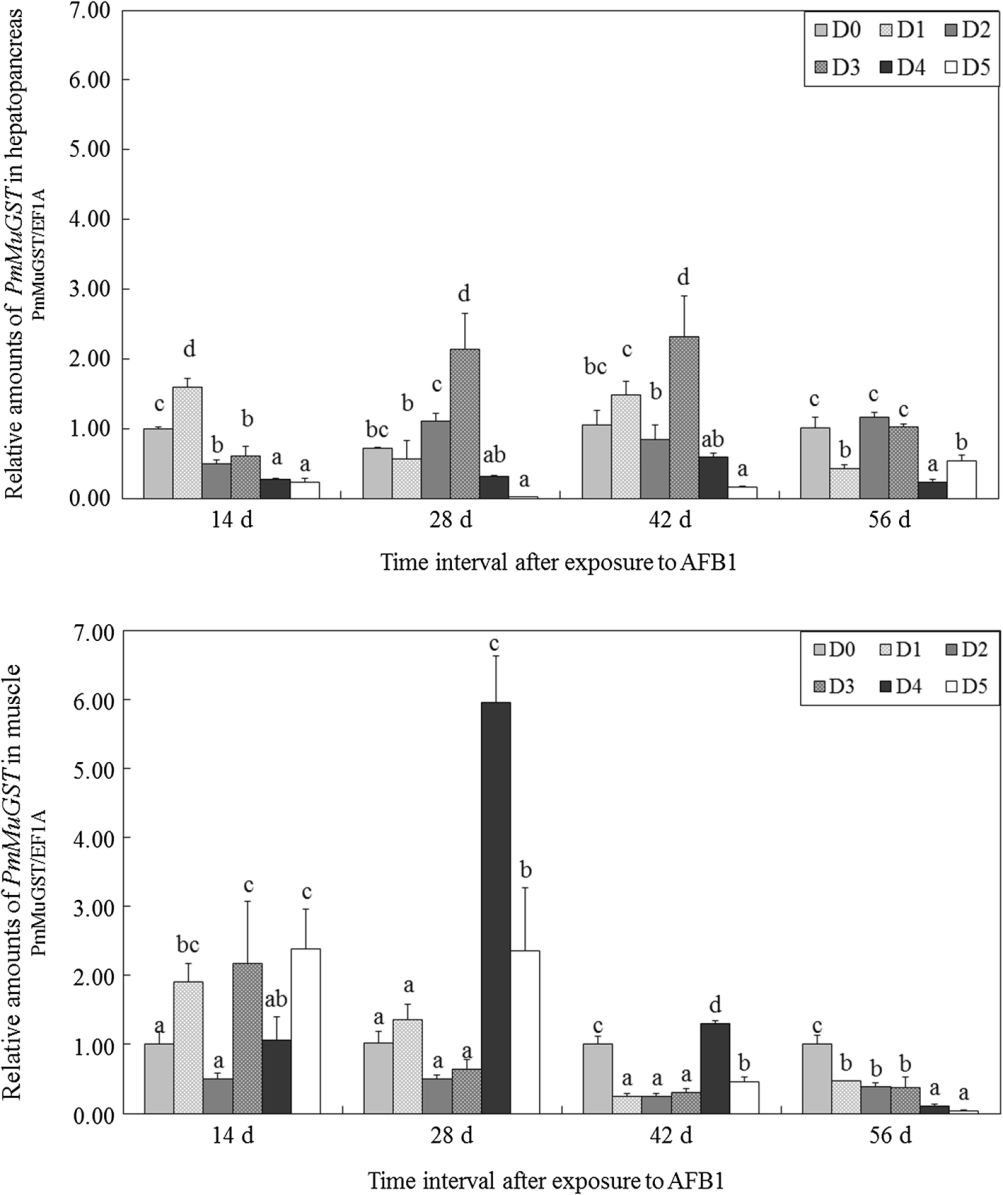
The transcript levels of *PmMuGST* mRNA in the hepatopancreas and muscle of tiger shrimp after treatment with different dosage of AFB1. Hepatopancreas and muscle of tiger shrimp fed with experimental diets were collected from nine individual tiger shrimp (three shrimp from each tank) at different time points. The expression values were normalized to elongation factor 1-alpha (EF1A) expression using the relative standard curve. *Each bar* represents the mean fold-change value from three tiger shrimp with the standard deviation (mean ± SD, *n* = 3). The *error bars* represent the corresponding SD from triplicate trials. The *different lowercase superscripts* represent significant differences in pairwise comparisons with control group (*P* < 0.05)

The transcript levels of *PmMuGST* in the hepatopancreas of shrimp decreased with the increase of AFB1 level (0–2500 μg/kg), while increased with the increased AFB1 exposure times (14–56 days) (Fig. 6a). Analysis of variance indicated there was significant interaction between the effect of AFB1 dose and exposure time on *PmMuGST* mRNA expression (*P* < 0.05) (Table 5). *PmMuGST* mRNA transcript levels in the hepatopancreas of D5 group were significantly lower than those of D0 group after exposure to AFB1 from 14 to 56 days. *PmMuGST* transcript levels of D4 group were significantly lower than that of D0 group at 14 and 56 days. The transcript levels of *PmMuGST* in the hepatopancreas of D3 group were significantly higher than that of D0 group at 28 and 42 days (Fig. 6a).

**Table 5:** ANOVA table of *PmMuGST* mRNA expression levels in the hepatopancreas and muscle and PmMuGST protein levels in the hepatopancreas of *P. monodon* after AFB1 exposure at 14, 28, 42 and 56 d (time, T) at different AFB1 concentrations (A)

Source	df	Sum of squares	Mean square	F	Pr > *F*
*PmMuGST* mRNA levels in the hepatopancreas (PmMuGST/EF1A)					
Corrected Model	23	24.710	1.074	28.467	0.000
A	5	13.350	2.670	70.751	0.000
T	3	1.550	0.517	13.695	0.000
A × T	15	9.809	0.654	17.327	0.000
Error	48	1.811	0.038		
Total	72	75.711			
*PmMuGST* mRNA levels in the muscle (PmMuGST/EF1A)					
Corrected Model	23	107.151	4.659	39.906	0.000
A	5	19.343	3.869	33.138	0.000
T	3	30.156	10.052	86.104	0.000
A × T	15	57.652	3.843	32.923	0.000
Error	48	5.604	0.117		
Total	72	202.642			
PmMuGST protein levels in the hepatopancreas (PmMuGST/GAPDH)					
Corrected Model	23	715.587	31.112	719.779	0.000
A	5	98.396	19.679	455.274	0.000
T	3	379.652	126.551	2927.720	0.000
A × T	15	237.538	15.836	366.359	0.000
Error	48	2.057	0.043		
Total	72	1235.345			

*PmMuGST* transcript levels in the muscle of shrimp increased with increase of AFB1 concentration ranging from 50 to 500 μg/kg, and decreased with the increase of AFB1 concentration ranging from 500 to 2500 μg/kg. The mRNA levels of *PmMuGST* in the muscle decreased with increased AFB1 exposure times in the range of 14 to 56 days (Fig. 6a). Analysis of variance indicated that there was significant interaction between the effects of AFB1 dose and exposure time on *PmMuGST* mRNA expression levels (*P* < 0.05) (Table 5). The transcript levels of *PmMuGST* in the muscle of D5 group were significantly higher than that of D0 group at 14 and 28 days. *PmMuGST* transcript levels in D4 group were significantly higher than that of D0 group at 28 and 42 days. At the end of the experiment, the transcript levels observed in AFB1 treated groups were all lower than that of D0 group (*P* < 0.05) (Fig. 6a).

### Analysis of PmMuGST protein in the hepatopancreas of *P. monodon* after AFB1 exposure

**Fig. 7:**
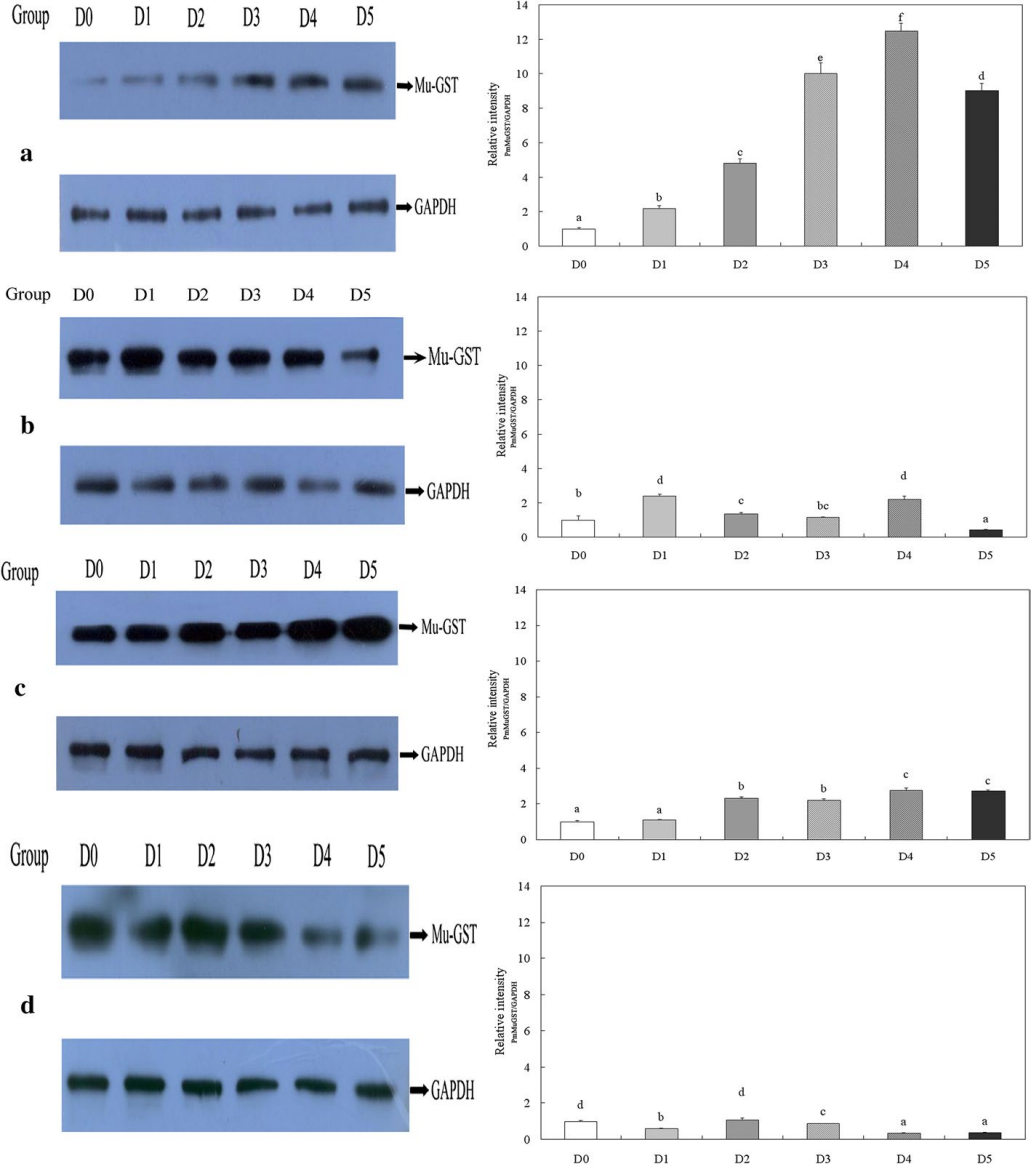
PmMuGST protein in the hepatopancreas of *P. monodon* at different time points after AFB1 exposure. PmMuGST protein was determined using western blot analysis with a *PmMuGST* antibody at 14, 28, 42 and 56 days post-exposure displayed in **a**–**d**, respectively. Equal amounts of proteins (30 μg) were subject to western blot analysis. GAPDH protein was used as an internal control. The amounts of GAPDH were also assessed to monitor the equal loadings of protein extracts. The optical density of the bands was determined directly on the film using IPP6.0 Analysis software. The relative intensity of objective protein was calculated based on the optical density ratio of PmMuGST and GAPDH bands. The *different lowercase superscripts* (*a*–*f*) represent significant differences in pairwise comparisons with control group (*P* < 0.05)

PmMuGST protein levels in the hepatopancreas of shrimp increased with increase of AFB1 concentration from 0 to 2500 μg/kg, and decreased with increased AFB1 exposure times in the range of 14 to 56 days (Fig. 7). Analysis of variance indicated there was significant interaction between the effects of AFB1 dose and exposure time on PmMuGST protein levels (*P* < 0.05) (Table 5). At day14 the levels of PmMuGST protein in the hepatopancreas of shrimp treated with AFB1 were significantly higher than that of the control group (*P* < 0.05) (Fig. 7a). The levels of PmMuGST protein in the D1, D2 and D4 group at 28 days were significantly higher than those of the D0 group (Fig. 7b). After AFB1 exposure for 42 days, the PmMuGST protein levels of shrimp in the groups treated with AFB1 (100, 500, 1000 and 2500 μg/kg) revealed significantly higher than that of the control (Fig. 7c). At the end of the experiment, the levels of PmMuGST protein in the D1, D3, D4 and D5 groups were all lower than that of the D0 group (*P* < 0.05) (Fig. 7d). In sum, the results demonstrated that the PmMuGST protein level was changed after AFB1 exposure.

## Discussion

In the present study, a Mu-class GST with a full-length cDNA sequence of 867 bp was cloned from the hepatopancreas of *P. monodon*. The cytosolic GSTs in most organisms are all dimeric with subunit molecular mass varied from 21 to 29 kDa (Mannervik and Danielson 1988). Our predicted molecular weight of *PmMuGST* was consistent with previous study which reported a Mu class GSTs with an average of 26 kDa of subunit molecular mass in human and rat (Blanchette et al. 2007). GSTs have two classical ligand-binding sites (G and H) that mediate binding of glutathione and electrophilic xenobiotic substrates to GST, respectively. The G site contains eight highly conserved amino acids: Tyr^7^, Trp^8^, Trp^46^, Lys^50^, Asn^59^, Leu^60^, Gln^72^ and Ser^73^, which is highly specific for glutathione, is conserved among all GSTs. For most of Mu-GST, Tyr^7^ is the key residue in the G-site. Site-directed mutagenesis studies of the Mu-GST revealed that Tyr^7^ residue plays an essential role in GST catalysis in promoting and stabilizing the thiolate anion (Wilce and Parker 1994). However, Contreras-Vergara et al. (2008) recently reported that Tyr7 is not critical in *L. vannamei*, as its mutant has 40 % of the wild type catalytic efficiency. Mu-loop, exclusive to Mu-class GSTs, is an insertion in the sequence compared to other classes of GST (Dirr et al. 1994) and is also a characteristic feature of Mu-class GST. The Mu loop in *P. monodon* including seven amino acid residues is consistent with the Mu-class GST in *T. clavigera* (Rhee et al. 2008), which usually occurs in other Mu-class GSTs (Dirr et al. 1994). The last amino acid residue of Mu loop in *P. monodon* was Asp, which is the same as that in the most other species. BLASTP results showed that the deduced amino acid sequence of PmMuGST had high similarity (86 %) with Mu-GST of *L. vannamei*. The sequence alignment and phylogenetic analysis further suggested that *PmMuGST* was a member of the Mu-GST family.

The tissue distribution of *PmMuGST* mRNA, as well as its response to AFB1 expousure, was investigated. In crustaceans, the distribution of *MuGST* in different tissues has been investigated in a variety of organisms, but the results are not similar. The amount of Mu-class GST mRNA is high in the hepatopancreas and gills in white shrimp of *L. vannamei* (Contreras-Vergara et al. 2004) and is high in gill in rock shell *T. clavigera* (Rhee et al. 2008). High amounts of Mu GST mRNA were found in the gills and gonad of disk abalone (Wan et al. 2008b). High transcript levels of *GSTs* in the hepatopancreas have been reported in fish and mollusk species (Gallagher et al. 1995). The *PmMuGST* mRNA was detected in all of the examined tissues, and the amounts of *PmMuGST* mRNA in the hepatopancreas and muscle were higher than those in other tissues, which was similar with the reports in *L. vannamei* and fish (Contreras-Vergara et al. 2004; Gallagher et al. 1995). Interestingly, the obviously high mRNA expression level of *PmMuGST* was found in the key metabolic center tissues including hepatopancreas and muscle, which suggested the role of PmMuGST in detoxification process.

The results of this experiment indicated that there were no notable differences in survival among shrimp exposed to different AFB1 concentrations ranging from 50 to 2500 μg/kg feed throughout the feeding period of 56 days. Boonyaratpalin et al. (2001) reported a similar finding in *P. monodon* that diets containing AFB1 at a dosage of 50 to 1000 μg/kg did not cause serious death in shrimp for 56 days. Wang et al. (2012) even concluded that diets containing 400–1600 μg/kg AFB1 did not increase mortality in *L. vannamei*. And our study indicated that the black tiger shrimp could tolerate a high dose of dietary AFB1. However, WG of *P. monodon* in AFB1 treated groups decreased in the present study. And the similar reports were found in *P. monodon*, and *L. vannamei* (Boonyaratpalin et al. 2001; Wang et al. 2012). However, we found that WG (185–611 %) in those previous experiments was much higher than that of WG (90–320 %) in the present study, suggesting that the initial body weight and different species or other environmental factors were involved in experiments and strengthened the toxic effects of AFB1.

The transcript pattern of *PmMuGST* was slightly different between the hepatopancreas and muscle, which might be related to the different functions of these tissues in response to the AFB1 exposure. In the present study, *PmMuGST* transcript levels in the hepatopancreas of shrimp decreased with increased AFB1 concentration. Boonyaratpalin et al. (2001) observed that severe degeneration of hepatopancreatic tubules was common in shrimp fed with high concentrations of AFB1 for 8 weeks, as noted by atrophic changes, followed by necrosis of the tubular epithelial cells. Therefore, it was understood that AFB1 toxic effects likely caused the histological changes and severely impaired the normal function of the shrimp’s cells, which might be the reason for the reduction of *PmMuGST*. Moreover, PmMuGST mRNA levels in the hepatopancreas of shrimp increased with AFB1 exposure time. The similar result was reported in *P. martensii*, that *PmMGST3* mRNA levels in the hepatopancreas increased to 2.4-fold of the control after exposure to cadmium for 3 days (Chen et al. 2011). However, the transcript level of GSTM (Mu-class GST) in the hepatopancreas of *C. cahayensis* and the activity of GST in the hepatopancreas of *R. philippinarum* were significantly decreased after exposure to a high level of microcystin-LR (100 μg/L) and cadmium (40 μg/L) (Li et al. 2014; Zhang et al. 2013). The transcript profiles of GST mRNA in response to AFB1, cadmium and microcystin-LR exposure indicated that it was inducible and might play an important role in the detoxification response in *P. monodon*.

In crustaceans, the hepatopancreas is the key metabolic center for the production of reactive oxygen species (ROS) (Duan et al. 2015). The hepatopancreas also plays a major role in the immune defenses of crustaceans (Söderhall and Cerenius 1998), and it is involved in both the synthesis of digestive enzymes and the detoxification of oxenobiotics (Vogt 1994). Therefore, we investigated PmMuGST protein levels by western blot in the hepatopancreas of shrimp exposed to AFB1 exposure. The results showed that PmMuGST protein levels increased with increase of AFB1 concentration from 0 to 2500 μg/kg. It may be that PmMuGST mitigates the toxic effects of AFB1 and/or neutralize harmful free radicals generated by AFB1. In lymphocyte-rich mononuclear cells, AFB1 exposure causes the production of ROS and causes biomolecular oxidative damage in broiler lymphocytes (Zimmermann et al. 2014). However, PmMuGST protein levels decreased with increased AFB1 exposure time in the range of 14–56 days, which might related to the lower growth and survival of shrimp. Nevertheless, it was clear that the patterns of *PmMuGST* mRNA transcript shown in Fig. 6 were seemingly uncorrelated well with the abundance of PmMuGST protein shown in Fig. 7. The absence of mRNA–protein correlation for a subset of investigated genes suggested that the relation between mRNA and protein was not strictly linear but has a more intrinsic and complex dependence, deviating from the classical view referred to as the molecular dogma. Different regulation mechanisms (such as synthesis and degradation rates), acting on both the synthesized mRNA and the synthesized protein, affect the amount of the two molecules differentially. Moreover, the protein translation usually lags the mRNA expression of genes, and the degradation rate of protein is much lower than that of mRNA; thus, there are also cumulative effects. We believe these may be the reasons for the absence of a correlation between mRNA and protein expression.

## Conclusion

In summary, a full-length cDNA sequence of a Mu-class GST was cloned from the black tiger shrimp *P. monodon*, and it was constitutively expressed in the following tissues: hemocytes, hepatopancreas, muscle, heart, ovary, stomach, eyestalk, and intestine. The expression of *PmMuGST* in the hepatopancreas and muscle changed dynamically in response to AFB1 exposure, which indicated that *PmMuGST* was inducible and was involved in the response to AFB1 exposure.
